# P10 Collapse, confusion and acute kidney injury presenting in the context of bullous skin disease in a young male patient

**DOI:** 10.1093/rap/rkad070.031

**Published:** 2023-09-27

**Authors:** Deepak Nagra, Christopher Baldwin, Jonathan Dick, James Galloway, Aveen Connolly, Chris Wincup

**Affiliations:** King's College London, Centre for Rheumatic Disease, London, United Kingdom; King's College Hospital, Dept of Rheumatology, London, United Kingdom; King's College Hospital, Dept of Rheumatology, London, United Kingdom; King's College Hospital, Dept of Renal Medicine, London, United Kingdom; King's College London, Centre for Rheumatic Disease, London, United Kingdom; King's College Hospital, Dept of Rheumatology, London, United Kingdom; King's College Hospital, Dept of Dermatology, London, United Kingdom; King's College Hospital, Dept of Rheumatology, London, United Kingdom

## Abstract

**Introduction:**

Cutaneous manifestations of systemic erythematous lupus (SLE) are common and reported to be present in 75-80% of patients at some point during the course of their illness. Further, lupus-related skin conditions can be the presenting symptom of the disease in a quarter of cases. Bullous skin changes are reported in SLE but are rare, affecting fewer than 5% of patients. Herein, we present a case of SLE in a young male in which bullous skin disease was the index symptom of a severe and rapidly evolving presentation associated with multi-organ involvement that was successfully treated with cyclophosphamide.

**Case description:**

A previously fit and well 17-year-old male of Caribbean ancestry initially presented with acute onset widespread bullous skin lesions distributed on his arms, legs, face and torso. Biopsy of these lesions revealed linear IgA deposition in keeping with SLE-related bullous skin disease. He was initially managed with a short course of oral prednisolone resulting in some transient improvement in his symptoms.

Twelve months later, he presented acutely to hospital following an episode of collapse at home. On arrival at hospital, he had evidence of widespread bullous disease with some new active lesions. Examination also revealed clinical evidence of a moderate sized right sided pleural effusion and inflammatory synovitis in bilateral knees. He appeared acutely confused and was disorientated for the first three days of admission. Investigations revealed an acute kidney injury on with a serum creatinine of 150 µmol/L, an elevated urine protein: creatinine ratio (126) and a low serum albumin (26 g/L). DAT was positive and a blood film suggested haemolysis. Echocardiography confirmed the presence of a moderate pericardial effusion. A CT brain was suggestive of inflammatory meningeal disease and subsequent lumbar puncture demonstrated an elevated protein (1.15 g/L) with a marked lymphocytosis. Testing for was tuberculosis was negative. Immunology confirmed a strongly positive anti-nuclear antibody (ANA, 1:1280), anti-dsDNA titres >400 units with a low complement C3 (0.35 mg/dl) and C4 (0.05 mg/dl). Renal biopsy demonstrated class III lupus nephritis.

A diagnosis of SLE was made with bullous cutaneous disease present in the context of active disease in the neurological, respiratory, cardiac, articular, haematological and renal domains confirmed (SLEDAI-2K = 28). Initial management with intravenous methylprednisolone resulted in a rapid improvement in cognition. A tapering course of oral prednisolone in combination with EUROLUPUS cyclophosphamide was initiated with an excellent clinical and serological response.

**Discussion:**

In this case, bullous skin lesions were the earliest indicator of SLE that progressed to rapid multi-organ involvement with extra-cutaneous manifestations including lupus meningitis, acute confusional state, pericardial and pleural effusions, autoimmune haemolysis, inflammatory arthritis and proliferative lupus nephritis. The initial presentation raised concerns about a potentially infective meningoencephalitis and initial management with antimicrobial agents was started. Following discussion with the wider MDT (including dermatology, nephrology, neurology and infectious diseases) and upon confirming the diagnosis of SLE, IV methylprednisolone was commenced. A rapid improvement in neurological/cognitive symptoms was observed, with the patient immediately becoming reorientated within 24 hours of beginning steroid therapy. Further therapy required careful consideration as therapeutic options effective in suppressing active disease across a number of domains was required. There are currently few evidence-based recommendations for the management of bullous lupus and a variety of agents were considered. The agreed MDT consensus was for the introduction of EUROLUPUS regime cyclophosphamide on the basis of concurrent proliferative lupus nephritis and cerebral involvement. Mycophenolate was added on completion of cyclophosphamide and oral prednisolone doses were gradually weaned. The patient has remained well to date and tolerated therapy without complication. Clinical progress is summarised in Table 1.

**Key learning points:**

The clinical heterogenicity of SLE fuels the delay in diagnosis, with the average time from first symptom to a confirmed diagnosis in the UK currently 7 years. Cutaneous manifestations of the disease are common and frequently observed as an early indicator of the diagnosis. Whilst cutaneous lupus can occur in isolation, bullous disease is typically associated with systemic disease. This case highlights the need to assess individuals for systemic involvement when presenting with isolated cutaneous bullous lesions. In this case, baseline immunological testing (including ANA, anti-dsDNA and complement) were not performed at the time of his initial presentation to dermatology. We suggest that the presence of bullous skin disease in the context of either serology or other clinical features of SLE should prompt an urgent referral to rheumatology. Management of bullous lupus is based upon small cases series with very few of this cutaneous manifestation included in large randomised controlled trials. Recommendations suggest the possible efficacy of dapsone or systemic immunosuppressive agents such as azathioprine and mycophenolate. Newer therapies such as anifrolumab have been successful in treating cutaneous lupus; however, they are currently not licenced in the UK. Multiple JAK inhibitors are currently under investigation in phase 2 and 3 trials for use in SLE, and there are case reports of the effective use of tofacitinib in bullous lupus. In this case, we demonstrate rapid improvement of SLE disease activity over a number of domains using EUROLUPUS cyclophosphamide therapy. However, there was an episode of minor reoccurrence of localised active bullous lupus (confirmed on repeat biopsy) after the third dose of cyclophosphamide that improved following a temporary increase in oral prednisolone dose. This demonstrates that bullous also has the potential to break through therapy that is otherwise controlling systemic manifestations but responds rapidly to increased steroid treatment.

**Table:** P10 Table

	Baseline diagnosis of bullous skin disease	Hospital admission with multi-system flare	2-weeks post initiation of therapy	4-weeks post initiation of therapy	8-weeks post initiation of therapy	12-weeks post initiation of therapy
Anti-dsDNA antibody titres	N/A	>400	>400	>400	115	
Complement C3	0.51	0.35	0.48	0.48	0.57	0.96
Complement C4	0.05	0.02	0.02	0.05	0.05	0.17
Urine PCR	N/A	1293	613	593	317	26.5
SLEDAI-2K	N/A	28	28	18	13	8
Cumulative therapy to date	20mg prednisolone	IV methylprednisolone 500mg x 3	60mg prednisolone + cyclophosphamide	40mg prednisolone + cyclophosphamide	20mg prednisolone + cyclophosphamide	Hydroxychloroquine 200mg

